# Cynicism and other attitudes towards patients in an emergency department in a middle eastern tertiary care center

**DOI:** 10.1186/s12909-016-0539-y

**Published:** 2016-01-29

**Authors:** Nicholas J. Batley, Zeina Nasreddine, Ali Chami, Dina Zebian, Rana Bachir, Hussein A. Abbas

**Affiliations:** Department of Emergency Medicine, American University of Beirut, Beirut, Lebanon; Current Address: Department of Molecular and Cellular Oncology, University of Texas M D Anderson Cancer Center, Houston, TX 77030 USA

**Keywords:** Cynicism, Empathy, Medical students, Interns, Residents, Attending physicians, Patients

## Abstract

**Background:**

A caring, compassionate practitioner of the medical arts is the idealized version of what makes a good doctor. If asked to think of a painting of a doctor we most likely conjure an image of a physician sitting at a patient’s bedside checking the pulse with a concerned look on his face. The reality is however that cynicism, among other negative attitudes, is becoming more prominent among physicians and medical staff. The causes and extent of cynicism likely vary among medical departments and different cultures. In this study, we aimed to assess attitudes of medical students and physicians in an Emergency Department (ED) in Lebanon that accommodates both local patients and is also known to attract patients from around the Middle East.

**Methods:**

A total of 30 students, residents and attending physicians at the American University of Beirut Medical Center were invited to participate. All participants underwent semi-structured interviews that were recorded, transcribed and then analyzed for common themes.

**Results:**

More negative emotions were expressed among participants than positive ones. Negative emotions were more frequently expressed among medical students, interns and residents than attending physicians. Cynicism in the ED was commonly reported however, maintenance of professionalism and adequate patient care were underscored. While empathy was recurrently found among participants, a trend towards a decrease in empathy with career progression was noted among attending physicians. Further, negative feelings towards patient families were prominent. Participants tended to categorize patients based on willingness to cooperate, gender, age, case acuity, ethnic origins and social status.

**Conclusions:**

Cynicism emerged as a prominent theme among medical students and staff in our study. However, participants were also empathetic. These attitudes were generally attributed to the peculiar stressors associated with the Lebanese culture, low acuity cases and “VIP” patients. It is crucial to explore methods in order to decrease cynicism and improve patient care. Also, the implications of these attitudes on patient care remain to be discovered.

**Electronic supplementary material:**

The online version of this article (doi:10.1186/s12909-016-0539-y) contains supplementary material, which is available to authorized users.

## Background

Cynicism is the tendency to doubt and disbelieve the sincerity of strong moral principles and motives [1, 2]. Attempts to tackle cynicism and promotion of a professional attitude towards patients via medical education are underway [3]. Cynicism can lead to burnout, derogatory humor and a significant decrease in empathy towards patients [3–5]. Among medical staff there has been a troubling inclination towards cynicism and a significant decline in empathy towards patients [6]. An attitude of cynicism is also common among medical students [6, 7]. Several studies found that medical supervisors failed at being role models in teaching how to establish a good relationship with patients and lacked required humanistic characteristics [8, 9]. These notions were further supported by the finding of Crandall et al., who showed that fourth year medical students are less inclined to care for underserved patients than first year medical students [10].

A factor that might temper the effects of cynicism may be empathy, which is defined as a cognitive attribute that involves an understanding of a person’s experiences, concerns and perspectives combined with a capacity to communicate this understanding [11]. When dealing with patients, empathy is crucial for wellbeing and contributes significantly to the alleviation of a patient’s stress and anxiety (West et al., [12]). Evidently, empathy significantly improves the patients’ outcomes and compliance as exemplified in diabetic and other patients [13–15].

There has however been a significant decline in empathy among medical staff with several factors contributing to this trend, including personal experiences, ethnic/racial prejudices, stereotypes, physical appearance, stress, anger, frustration, exhaustion, work environment and job satisfaction [16–18]. There is also an increasing tendency for patients to visit the emergency department (ED) for non-urgent conditions that may increase the workload for medical staff leading to more frustration and burn out [19]. Root cause analysis can identify other factors that could be prevented or ameliorated in order to decrease cynicism and improve empathy. To that end, two models were suggested that might lead to cynicism among medical students. The “professional identity” model suggests a temporary cynicism while the student is developing until they reach a certain position of authority in the health care system [2]. The other model is dubbed the “intergenerational transmission” where cynicism is learnt from higher ranking peers; the hidden curriculum of medical school [2].

The trend of developing cynicism among medical staff contrary to their expected role of being compassionate and empathetic can be referred to as “traumatic de-idealization” [20]. Nevertheless, the extent of traumatic de-idealization may vary among different medical settings and cultures. The Emergency Department at American University of Beirut Medical Center accommodates patients from various ethnic and social backgrounds. The purpose of this study is to explore the attitude of medical students, trainees and staff towards patients in the emergency department, specifically looking at the negative emotions generated by patient encounters. Simultaneously, this study explored the attitudes toward patients and their families. Findings from this study can address whether or not interventions are warranted in order to modify the physician’s attitudes.

## Methods

### Setting

This study was conducted at the Emergency Department of the American University of Beirut Medical Center between the years 2011 and 2013. All participants had spent at least three weeks working in the Emergency Department of American University of Beirut Medical Center during the study period.

### Ethics, consent and permissions

Institutional Review Board (IRB) approval (ID: ER.NB.03) at American University of Beirut Medical Center was received prior to initiation of the study on November 16, 2011. The IRB committee at American University of Beirut Medical Center ensures full protection of human subjects. All participants were informed about the goals and expectations of this study and were informed of their right to withdraw from this study at any point. All data and safety monitoring were in compliance with the Protection of Human Safety regulations.

### Data collection

The sample consisted of 11 medical students (6 fourth year and 5 third year – in a four year program), 6 interns, 7 residents, and 6 attending physicians for a total of 30 participants (60 % males and 40 % females) with an average age of 27.5 years. All participants signed a consent form explaining the study details and participants’ rights to withdraw from the study at any point. Semi-structured questions were developed and interviews were conducted over 30–40 min. While there was no specific criterion for data saturation measurement, we stopped recruiting new participants when the input from participants became significantly redundant and no new codes were emerging. Since the primary language of teaching and training at American University of Beirut Medical Center is English, all interviews were conducted in English by Lebanese staff who have proficiency in spoken and written English. (Additional file 1). Recordings of the interviews were transcribed by two people (Medical Degree (MD) and Masters in Public Health (MPH) holders). Our methodology adheres to COREQ guidelines for reporting qualitative analysis (Additional file 2).

### Data analysis

Thematic Content Analysis (TCA) was used to analyze 30 interview transcripts and was conducted by a research member who was not involved in the interview process. TCA is a descriptive presentation of qualitative data aimed at identifying common themes in texts provided for analysis [21]. Although a priori themes (deductive approach) were identified for extraction prior to data coding (cynicism in the ED, low acuity patients, emotions in the ED), the interview process was not uniform across different participant groups, hence open coding or an inductive (bottom-up) procedure was used, where themes were extracted from the data as they occurred [21–23]. The unit of analysis that was coded was chunks of text; this hence reduced the amount of data.

Analysis of the interview data involved four phases. First the interviews were transcribed by two people and checked for accuracy. Second, five transcribed interviews were selected at random, one from each of the five groups of participants, namely, attending physicians, residents, interns, fourth year and third year medical students. These interviews were subject to an initial screening process by two independent coders highlighting descriptions relevant to the study aims. Each highlighted unit of text was ascribed a distinct meaning or code. These codes were then discussed and compared across the two coders to reach consensus on labels and meanings. Preliminary codebooks were generated for attending physicians (nine codes), third year medical students (one code), fourth year medical students (one code), residents (ten codes) and interns (five codes).

In phase three, the two coders read and coded the rest of the transcribed interviews, meeting frequently to compare coded units and reach consensus. Throughout the process, emerging codes not present in the initial codebook were added and updated. At this stage, ten codes were extracted for interns, nineteen for residents, sixteen for third year medical students, nineteen for fourth year medical students, and twenty two for attending physicians.

After completion of coding, similar codes were identified across the five groups of participants for common themes. Each theme included a class of subthemes. Analyses were based on the frequency of comments made which is the number of times comments were made regardless of the number of people making them, as well as the extensiveness of comments which is the number of people who made the comments.

## Results

### Cynicism and empathy

The responses of participants were assessed for cynicism and empathy and recurrent themes were found among participants although differences were also deciphered.

### Cynicism in the ED

Cynicism in the ED, defined as the inclination to believe that patients coming to the ED are motivated by selfish interests rather than actual emergencies, was reported to be common by all participants. Three fourth year medical students and one third year medical student made a total of 5 cynical comments in relation to low acuity patients in the ED. One of the fourth year medical students stated that low acuity patients do not present with actual emergencies and attend the ED mostly on Mondays and after holidays to get a sick leave.

“Mainly for fun…mainly after vacation and holidays and on Mondays…”

Fourth year medical student

The other two fourth year medical students stated that low acuity patients exaggerate their symptoms and are malingering, respectively. Finally the third year medical student stated that low acuity patients attend the ED because it is convenient compared to booking an appointment with their physician at his/her clinic.

Cynical comments by students were often followed up by self-regulatory statements buffering against behaviors that may harm patients. One fourth year medical student stated “but then it becomes another regular case…you never know what happens maybe there is something hidden or the patient is not describing accurately the situation…at the end you don’t want to miss anything…it is the ED at the end you never know…” - fourth year medical student, and another stated “… you always have to assume that it's a serious complaint and go through the hoops as such”. Similar self-regulatory statements were made by interns (*n* = 1), residents (*n* = 4) and attending physicians (*n* = 8). These statements were often framed as recommendations to avoid behaving out of cynicism. One attending stated “Patients do not always have the know how to deal with non-urgent but concerning cases” another recommended the adoption of a “what if” attitude. Most of all, maintaining professionalism in patient care was used as a self-regulatory strategy when dealing with low acuity cases.

No cynical comments were made by attending physicians, interns and residents, nevertheless various sources of cynicism were identified including how busy the ED was at a given time, the patient’s family members, the overall environment of the ED and its urgency, and doing someone else's job.

Cynicism was also attributed in part to the Lebanese culture, which is troubled by significant political and economic instability, and in part to a lack of training.

“Everyone has cynicism…The cynicism is related to how busy you are. You rarely find a physician who has cynicism when he has nothing to do…It is either bad time for you or you are really busy and this patient is wasting time…”

Attending physician

“I'm seeing that a lot of attending physicians and residents are mostly cynical, nobody is altruistic. No one is completely altruistic; I mean there's a percentage, like someone is 70 % altruistic and 30 % cynical…At first, I was annoyed by some people here who are really cynical, but later on I noticed that they're actually still efficient, so now I don't mind the negative atmosphere…”

Third year medical student

Cynicism was reported to be higher among residents compared to students by an attending physician. Participants identified cynicism as a factor that can negatively influence patient management. Yet, one medical student asserted the following: “there is no excuse for not taking full care of the patient regardless of how much they exaggerate or overstate their symptoms" (Med 3).

Some attending physicians were reported to act respectfully with low acuity case while others were reported to be dismissive, and not giving enough time and explanation. One student reported that behavior toward patients by attending physicians was "by the book" (Med 4) and detached as if they were faking it. On the other hand, attending physician behavior towards patient family members was reported to be beneficial for students in terms of learning how to address difficult family member encounters.

### Empathy

Empathy towards patients was a recurrent theme among attending physicians, residents and fourth year medical students. Empathy was reported to be higher when dealing with children and the elderly.

“More empathetic with pediatric cases…Having kids is different…I didn’t use to empathize as such when I didn’t have kids. Also, I empathize more with elderly”

Attending physician

A crucial aspect of empathy as reported by an attending physician was the ability to relate to the patient. However the need to maintain a healthy balance of empathy and distance between a patient and a physician was stressed by one of the medical students:

“There is a limit. You have to be empathetic, but you cannot be too empathetic, and again, this should come with experience; to know at which point you should stop being too involved with the patient, and at which point to engage more…”

Fourth year medical student

Another emergent subtheme was the reported decrease in empathy over the course of one’s medical career from student to attending physician as a function of exposure and burnout. Lack of empathy in a physician was reported to influence patient retention rate and not patient care, which is a situation that is more relevant to the clinic setting rather than the ED. Empathy was also reported to be influenced by how busy the ED is at a given time:

“Putting yourself on the side of the patient…Understanding the situation of the patient why he is acting like that. Maybe in the clinic I would have more time to do that but in the ER I don’t have time. I have noticed I’m more caring when the ER is empty and I have one patient I am more social I’m more caring and I give more but usually when the ER is busy I am just doing my job…”

Resident

We further analysed the transcripts for recurrent themes emerging from all participant groups. Three themes were common across all five classes of participants: patients, low acuity cases and patient family members (Table 1).

**Table 1:** Frequency and extensiveness of themes common across all participant groups (*N =* 30)

	Med3 (*n* = 5)	Med4 (*n* = 6)	PGY1 (*n* = 6)	PGY2 (*n* = 7)	Attending (*n* = 6)	Total
Patients	7	12	14	19	11	63
Low acuity cases	5	5	6	5	4	25
Patient family members	3	2	3	3	1	12

### Patients

A total of 14 patient related subthemes were identified. The most recurrent subthemes are presented in Table 2. Difficult patients were defined as patients who were uncooperative, entitled, nagging, egoistic, and had expectations for treatment that were well beyond what the institution should provide. A few interviewees expressed anger and irritation towards such patient encounters, while most did not have any specific negative or positive emotions. Understanding that difficult patients may be acting out of fear and being nonjudgmental were stressed as means of treating patients professionally and warding off negative feelings.

“…we see the snapshot and we tend to judge so I try not to judge because there is a story behind every patient.”

Resident

**Table 2:** Patient related subtheme frequency and extensiveness (*N =* 30)

Subtheme	Med4 (*n* = 6)	Med3 (*n* = 5)	PGY1 (*n* = 6)	PGY2 (*n* = 7)	Attending (*n* = 6)	Number
Difficult patients	2	2	3	3	3	13
VIP Patients	1		2	4	1	8
Preferred patients	2	1	1	3	3	10
Disliked patients	1		2	3	3	9
Patient attitude toward medical students	3	2				5

Very important person, or “VIP”, patients, those with connections with senior doctors in the institution, politicians, or the wealthy, were described as entitled, pulling strings and not caring for the lives of others. These patients were disliked by most participants (*N* = 8) as they generated feelings of anger, contempt and frustration.

“I want to add to the list of patients that I hate, the VIP patients; I hate them with passion and what makes them VIP is how the Attending behaves towards them.”

Fourth year medical student

One of the participants described them as “minefields” (intern) and explained that given their status, they attracted excessive attention from staff. Maintenance of optimal patient care was stressed although participants had a more distant and stiff attitude towards VIP patients.

Participants expressed an overall preference for working with patients that are cooperative, quiet and polite. Preference for specific groups of patients was also found namely, patients with higher education and socio-economic status, accident victims, disadvantaged patients, children, high acuity patients, and patients aged 4 to 50 years.

“It is always more thrilling to see a trauma, STEMI [heart attack], or [aortic] dissection patient compared to a case of simple upper respiratory infection. I have a slight preference in seeing a high acuity patient.”

Attending physician

VIP, disrespectful, and entitled patients were disliked. Nevertheless, interviewees confirmed that these preferences do not influence patient care but rather affect the frequency they are checked-up on during their stay in the ED and the extent of socialization with the patient. Further, participants preferred not to examine patients who were known frequent ED visitors.

The subtheme “patient attitudes towards medical students” was recurrent among third and fourth year medical students. While some reported that patients treat medical students as envoys and in a belittling and patronizing manner, others reported them to be positive, appreciative, apologetic and serious. When treated negatively some interviewees reported feeling spiteful while others reported being indifferent to the situation.

“I think we come in with the expectations that some of them [patients] are going to be this way, and we are indifferent to it. It happens in every rotation, maybe more often in the ED, but we know that this is the case.”

Third year medical student

### Low acuity cases

Low acuity cases were generally described unfavorably, such as being time consuming, a waste of time, meaningless, complaining, presenting with exaggerated symptoms, demanding and attention seeking. One of the third year medical students referred to them as interesting and educational. Noteworthy, attending physicians did not report to be bothered by low acuity cases.

“I am not bothered [by low acuity cases]. They don’t bother me. I think there is interesting stuff in the low acuity and they are not as stressed out and the high acuity is also interesting”

Attending Physician

However third and fourth year medical students reported mostly negative emotions (frustrated, annoyed, aggravated, angry, irritated, and unenthusiastic) with only one positive reference of being concerned. When probed, justifications for these negative feelings included cases not qualifying as actual emergencies, taking away from the learning experience as well as time to care for other patients, and being stuck doing paper work instead of medical work.

“The smaller - more trivial - the complaint, the more likely that the patient is going to be complaining. They tend to exaggerate or overestimate their symptoms versus when someone is actually having a serious complaint”

Fourth year medical student

“…she wasted my time…yeah it did irritate me especially that at other time when following up on that patient…it is irritating…because all what I did was paper work…”

Third year medical student

Interns and residents reported more negative feelings (angry, irritated, frustrated, upset, dissatisfied, and disappointed) towards low acuity patients, however an added dimension of how busy the ED may be was emphasized. Sources of negative feelings among interns and residents in relation to low acuity cases related to the patients’ behaviors (e.g. nagging, demanding to be admitted at once, not understanding hospital procedures, and bypassing the system) as well as beliefs that they took away from other urgent cases coming in at rush hours. Participants reported means of coping with these negative feelings by adopting a "you never know" attitude and getting rid of negative emotions by detaching from the situation. Occasionally, these cases were also referred to as comforting (easy cases) and anxiety free due to the lack of urgency. Participants also stressed the importance of maintaining a professional and ethical attitude towards the patient. Better triage was recommended as a means of avoiding having nonemergency cases end up in the ED.

“Low acuity cases such as gastroenteritis…can be treated at home…not necessary to get admitted to the ED…time consuming…”

Intern

### Patient family members

Patient family members were described as intrusive, interrogative, aggressive, entitled, disrespectful and having no situational awareness. The latter was attributed to the Lebanese culture which was described by one of the participants as hysterical, needy and demanding.

“There are cultural issues that are particular annoying. People have no sense of situational awareness so if they see you talking to another person so they come right up to you and interrupt you and listen in on the conversation with that patient…”

Attending physician

Only one participant among attending physicians addressed the positive aspects of having family members in the ER describing them as fun, hospitable and appreciative. Emotions generated from difficult encounters with family members were generally negative (frustration, stress, anger, hatred, disgust, irritation, contempt, feeling disrespected and underappreciated), and found least frequently among attending physicians.

“…Honestly I just wanted her to leave, so that was very frustrating, but it turned out that she knew my sister, and I couldn’t not deal with her. So what I tried to do was try to explain things to her…”

Third year medical student

Participants added that being abrupt by asking family members to wait brought the urgency of the ED to the family member's awareness and giving swift reassuring responses were effective means of dealing with patient’s family members who are oftentimes worried and anxious.

## Discussion

The current study aimed at qualitatively assessing the attitudes of medical staff in the ED and to investigate for sources of cynicism and empathy as expressed by students, interns, residents and attending physicians. While sources of cynicism and empathy emerged as prominent themes, additional themes were also extracted from the analysis and focused on patients, low acuity cases and patient family members.

### Cynicism

The presence of cynicism in the ED was acknowledged by participants, who identified inadequate distribution of tasks (task overload), patient family members, ED rush hours, lack of adequate training of physicians, and the Lebanese culture as sources of cynicism, among others. In line with our findings, task overload was identified as a strong significant predictor of cynicism along with role insufficiency in a study addressing burnout among physicians in China [24]. However, studies have not addressed the role of the interaction of patient family members in the development and maintenance of physician cynicism. Perhaps the added pressure of explaining treatment plans and managing patients’ immediate and extended family members within the Lebanese context, contributes to overloading physicians with additional tasks which would then lead to cynicism which has been identified as a sign of burnout [25]. Noteworthy, cynicism development may lead to decreased empathy towards patients [3–5]. Since empathy can significantly impact patient outcome, diagnostic accuracy and medical errors [3, 12–14], addressing the underlying causes of cynicism is warranted for better medical care. Although cynicism was reported by attending physicians to be highest among residents compared to medical students, it is expected to reduce as physicians progress in their medical careers. According to Testerman et al. [2] cynicism is highest among medical students and decreases as a physician moves forward in their medical career due to the development of coping skills [2]. It would be interesting to explore this hypothesis by interviewing the same medical staff at different stages in their career.

Further, a contributing factor to cynicism development could be the ED setting *per se*. Specifically, Campbell et al., argues that the “chaotic and ill-structured emergency department environment” contributes to cognition derailment while in the ED [26], and may ultimately lead to increased cognitive alterations such as cynicism. Similarly, Durning et al., emphasizes the significance of the *context* in assessing the clinical encounter [27]. Since ED encounters are generally short-lived and there is lack of ongoing relationships between the patients and clinicians, it is possible that this can also contribute to the negative feelings that are acquired compared to non-ED setting where a repertoire and a relationship is built between the medical staff and the patient. Considering these factors can establish a better foundation of underlying sources of cynicism among medical staff.

### Empathy

Empathy in the ED is influenced by numerous factors. For instance, the ED work load may contribute to the assessment of empathy as shown in a Taiwanese study [28]. Participants in the current study reported that empathy decreases as a physician progresses in their medical career. While trends of such a decrease has been previously reported [4], it has also been deemed as largely exaggerated and weak [29]. Participants stressed the detrimental role of a lack of empathy on patient care. In fact, over the past 20 years the roles of the therapeutic relationship has been established as an integral part of effective medical care [30].

### Patients in the ED

Our study indicated that ED staff found it emotionally difficult to interact with VIP status patients and difficult patients who are characterized as entitled and uncooperative. What characterizes a patient as difficult is more relevant to the emotions that a specific patient generates and how the physician deals with them [31]. It has been postulated that self-awareness in physicians is key to the prevention of such negative feelings influencing patient care [32]. Balint groups, where physicians can share their concerns, feelings and experiences have been shown to be effective in fostering self-awareness [33]. Participants in the current study asserted that stereotyping patients is wrong, yet they acknowledged the difficulty in avoiding this. Making an accurate and quick clinical judgment is considered an asset for physicians. However, when judgment extends to the patient’s sociocultural context, it becomes more of a liability than an asset [34]. Patient stereotypes have been shown to influence clinical care specifically for patients who abuse alcohol or drugs, are unhygienic, are abusive, and have minor mental disorders [35]. In the current study, the patients most stereotyped were young females who were labeled “hysterical” or as stated by participants “H Y S”.

### Low acuity cases

Different classes of participants referred to low acuity cases differently. While attending physicians expressed favorable attitudes towards low acuity cases, medical students, residents and interns were more negative when referring to these patients. The difference may be related to the decline in cynicism from being a medical student to an attending physician [2], or to the fact that attending physicians are less occupied with paper work as reported by participants in the study. In the current study low acuity cases were described as a waste of time and time consuming. This complaint was empirically assessed in a recent study only to show that even with cases deemed non-urgent, some form of medical care was still performed at the ED, calling into question the categorization of low acuity cases as unnecessary [36]. One of the sources of the negative attitude towards low acuity patients was related to a belief that they take away from the care of other more urgent cases. To the best of our knowledge, no empirical research addressing the matter within the Lebanese context has been published, however in a study examining ED waiting time as a function of low acuity cases, a negligible increase in patient waiting time was noted due to the presence of low acuity patients [37]. Such a finding indicates that all patients are receiving the care they seek with no delays to urgent cases as a function of low acuity cases. Future studies in the region can empirically assess this belief within the Lebanese/Middle East medical care context.

### Patient family members

Participants in our study found patient family members to be generally disruptive to the ER process and frustrating for staff and physicians. Patients in Lebanon tend to be accompanied by multiple family members. Although not quantified formally, it is not uncommon to have six to ten family members with a patient. Studies addressing the presence of family members in the ER have examined effects on patient wellbeing and have found that in general, the presence of family members has favorable outcomes for patients [38], specifically pediatric patients [39]. Future research needs to address the effect of family member involvement and presence in the ER on emergency staff in cultures where the extended family is a more prominent cultural phenomenon. Clearly, this was a major concern for the physicians in our study.

### Strengths and Limitations

Our study has included participants of different demographics, which allows for more in depth analysis based on rank and experience. We also attempted to understand the cultural context of the study, which allowed a more thorough understanding of the findings. While a set interview protocol was specified at the beginning of the study, within each group the interview protocol was slightly different. Another limitation was the presence of more than one and at times two interviewers per interview, which may have increased the number of clarifications per question thus increasing the likelihood of confusing the interviewee. Our study does not prove that that students and physicians who experience significant amounts of cynicism may provide poorer patient care. Since we did not collect any information regarding patients’ perceptions of empathy, we did not have sufficient evidence to link our findings to patients’ outcomes. Designing further studies, which attempt to uncover such a correlation would be an important step in improving the generalizability of our findings. Other significant limitations include the small sample size and the fact that this was a single center study; factors that further limit generalizability. This is however, the first such study of its kind in this setting and as such may be an important stepping-stone for future studies.

## Conclusion

This is the first study to assess the attitudes of medical staff in the ED in the Middle East. Our findings demonstrated cynicism in the ED but also empathy. Despite the negative emotions uncovered, participants emphasized appropriate patient care and professionalism. Further, attitudes towards patients, low acuity cases and family members were intriguing and included contempt, anger and frustration. An overall negative perspective towards patients was prominent among the participants. Although participants discussed some underlying causes that could have potentially led to these feelings, a root cause analysis for these outcomes is warranted. We believe that the findings of this study can be used to model future study designs where provider attitudes related to actual patient care could also be assessed.

Any attempt at reversing the trend towards cynicism starts with understanding the dynamics that create it. Our study highlights the negative emotions felt by medical staff in the ED, the degree of which suggests the need to acknowledge their existence and design training and educational strategies that will hopefully reduce them.

## Additional files

Additional file 1:
**List of the semi-structurered interview questions.** (DOC 32 kb) Additional file 2:
**COREQ questionnaire guidelines.** (XLSX 11 kb)

## Abbreviations

ED: Emergency Department
MD: Medical Degree
MPH: Masters in Public Health
TCA: Thematic Content Analysis
VIP: Very Important Person
