# Supplementary material for: Cynicism and other attitudes towards patients in an emergency department in a middle eastern tertiary care center
Source: BMC Med Educ. 2016 Jan 29;16:36. doi: 10.1186/s12909-016-0539-y (PMC4731937; doi:10.1186/s12909-016-0539-y)
Supplement: Additional file 1: — List of the semi-structurered interview questions. (DOC 32 kb) [file 12909_2016_539_MOESM1_ESM.doc]

**Semi-structured interview questions**

Good afternoon,

Thank you for taking the time to meet with us. We will honor your time by making sure that we wrap up in the next 30-60 minutes. As indicated in the consent form, the interview will be tape recorded to allow transcription and analysis of the data at a later stage. The data collected will be kept confidential; no one will have access to the data other than the principal investigator and the research assistant working on the study. Your participation is voluntary; you can withdraw at anytime during the interview. Any questions before we start?

1. What do you think of the cases admitted to the ED? Do they all represent ER cases?

*In case non-ER cases were encountered, a follow up question will be posed:* 2. How do you feel about these cases?

3. Based on your ED working experience, kindly describe the range of emotions and feelings you experienced during your encounters with patients…

*In case negative feelings were mentioned, a follow up question will be posed:*

4. Did you feel annoyed and irritated as a result of an encounter with a patient(s)? What are the reasons that made you feel this way?

5. Do you recall such an experience occurring with any other doctor in the ED?

6. Is role-modeling to guide avoiding negative feelings towards patients practiced in the ED? How?

**Focus group questions**

Good afternoon,

Thank you for taking the time to meet with us. We will honor your time by making sure that we wrap up in the next 60 minutes. This session will be tape recorded to allow for transcription and analysis of the data at a later stage. The collected data will be kept confidential; no one will have access to the data other than the principal investigator and the research assistant working on the study. Any questions before we start?

**Medical students, interns & residents**

- Please tell us about your working experience at the ED, particularly about your encounter with patients?
- Do you have preference for dealing with certain types or categories of patients over others?
- Are there any category/categories of patients that you would have preferred not to encounter at the ED?
- How do you feel about these different categories of patients admitted to the ED?
- Do you recall any scenarios of encounters with patients that provoked negative feelings in you or any of your colleagues?
- Please tell us more about such feelings?
- What caused the arousal of such feelings?
- Was such negative feelings communicated to the patient?
- What was the reaction of the patient(s) in consequence?
- Do you think that such feelings can compromise the quality of care received by patients? Please explain or give an example.
- How did you manage to deal with such encounters?
- Did you receive any guidance to help you get over with these negative feelings and feeling more positively towards patients?

**Attending physicians**

- Please tell us about your working experience at the ED, particularly about your encounter with patients?
- Do you have preference for dealing with certain types or categories of patients over others?
- Are there any category/categories of patients that you would have preferred not to encounter at the ED?
- How do you feel about these different categories of patients admitted to the ED?
- Do you recall any scenarios of encounters with patients that provoked negative feelings in you?
- Please tell us more about such circumstances?
- What caused the arousal of such feelings?
- How did you manage to deal with such encounters?
- Do you recall any scenarios of encounters with patients that provoked negative feelings in any of the medical team you were in charge of supervising at the ED?
- Please tell us more about such circumstances?
- What caused the arousal of such feelings?
- As a supervising physician, how did you manage to deal with such encounters?
- Do you make use of such situations to teach junior doctors how to avoid negative feelings and feel more positively towards patients?
- What is your teaching strategy?
- Was such negative feelings communicated to the patient?
- What was the reaction of the patient(s) in consequence?
- Do you think that such feelings can compromise the quality of care received by patients? Please explain or give an example.
